# Effect of orthokeratology on precision and agreement assessment of a new swept-source optical coherence tomography biometer

**DOI:** 10.1186/s40662-020-00177-4

**Published:** 2020-03-02

**Authors:** Bao Shu, Fangjun Bao, Giacomo Savini, Weicong Lu, Ruixue Tu, Haisi Chen, Benhao Song, Qinmei Wang, Jinhai Huang

**Affiliations:** 1 School of Ophthalmology and Optometry and Eye Hospital, Wenzhou Medical University, 270 West Xueyuan Road, Wenzhou, 325027 Zhejiang China; 2 Key Laboratory of Vision Science, Ministry of Health, Wenzhou, Zhejiang People’s Republic of China; 3 G.B. Bietti Foundation IRCCS, Rome, Italy

**Keywords:** Swept source optical coherence tomography, Orthokeratology, Precision, Agreement, Children

## Abstract

**Background:**

To evaluate the effect of orthokeratology on precision of measurements in children using a new swept-source optical coherence tomography (SS-OCT) optical biometer (OA-2000), and agreement between its measurements and those provided by the commonly used IOLMaster based on partial coherence interferometry (PCI).

**Methods:**

This study recruited fifty-one eyes of 51 normal children (8–16 years). An operator took measurements with the two biometers. Then, a second operator took measurements with the SS-OCT biometer. After orthokeratology was performed for one month, the same operators repeated the same procedures. Axial length (AL), mean keratometry (Km) at 2.5 mm and 3.0 mm diameters (Km_2.5_ and Km_3.0_), central corneal thickness (CCT), anterior chamber depth (ACD), lens thickness (LT) and corneal diameter (CD) were analyzed.

**Results:**

With the SS-OCT optical biometer, the test-retest repeatability of AL measurements was < 0.06 mm. For all parameters, the coefficients of variation were < 1.23% and the intraclass correlation coefficients were > 0.95. The 95% limits of agreement of difference between the two devices for CD parameter were up to 1.53 mm. After orthokeratology, the fluctuation ranges of difference for Km3.0 measurement was 1.11 times higher than before orthokeratology, while the absolute values of difference for AL, Km2.5, ACD and CD measurements were comparable.

**Conclusions:**

Before and after orthokeratology, the SS-OCT biometer showed high repeatability and reproducibility for all measurements. Wearing orthokeratology contact lenses affected the agreement between SS-OCT and PCI biometers for Km3.0 measurements. The CD measurement showed poor agreement between the two devices.

## Background

Orthokeratology involves wearing specifically-designed rigid corneal contact lenses, which can effectively correct low and moderate myopia [1] by reducing corneal curvature and retarding axial length (AL) growth [2]. As compared to frame glasses and soft corneal contact lenses, orthokeratology delays the growth rate of the eye axis in children from 32 to 55% [3, 4]. As a safe and efficient treatment for controlling myopia progression, orthokeratology is receiving increasing attention [5, 6].

When following-up with children undergoing orthokeratology, it is necessary to meticulously measure multiple ocular biometric parameters such as AL, corneal topography and endothelial cell count. As compared to stable corneal structure in adults, the corneal shape of children can easily deform in addition to a poorer fixation and shorter cooperation. Hence, the repeatability and reproducibility of ocular biometric measurements in children tend to be lower than in adults. However, the precision of measurements by ocular instruments in children and the influence of wearing orthokeratology contact lenses on the precision of such measurements have been rarely reported.

The new optical biometer OA-2000 (Tomey, Nagoya, Japan) applies swept-source optical coherence tomography (SS-OCT) technology to measure the optical distance between ocular structures [7]. It provides measurements of the AL, keratometry (K), anterior chamber depth (ACD), corneal thickness (CT), lens thickness (LT), pupil diameter (PD) and corneal diameter (CD). The optical biometer IOLMaster (Carl Zeiss Meditec AG, Jena, Germany), which introduced the partial coherence interferometry (PCI) technology, has been reported to show high precision of measurements in normal and cataract eyes, and is regarded as the gold standard for non-contact biometry [8, 9].

In contrast to the IOLMaster, the new SS-OCT biometer has automatic correction function and continuous measurement model. The new SS-OCT biometer was reported to have high repeatability and reproducibility in measuring normal subjects and cataract patients in a few studies [10, 11]. However, any precision changes in children wearing orthokeratology contact lenses have not been reported. Therefore, we used the new SS-OCT optical biometer and PCI optical biometer to measure ocular biometric parameters of children before and after orthokeratology in order to evaluate the intra-operator repeatability and inter-operator reproducibility of the new SS-OCT biometer, as well as agreement between the two instruments. This study is also the first to investigate whether orthokeratology influences the precision and agreement measurements of the new SS-OCT biometer.

## Patients and methods

### Patients

The study followed the tenets of the Declaration of Helsinki [12] and was approved by the Research Review Board of the Eye Hospital of Wenzhou Medical University (KYK2013–21). Before examination, we explained the procedures to the participants and their parents, and then informed consent was taken from all statutory guardians of the subjects. To decrease the impact of diurnal fluctuation on corneal structure, all subjects completed measurements between 09:00 am and 05:00 pm [13]. The inclusion criteria were as follows: age ≥ 8 years old, myopia ≥ − 5.00 D, astigmatism ≤ 1.50 D, normal intraocular pressure (10 – 21 mmHg), wearing the same orthokeratology lens and following the wearing time and follow-up plan, and having good fixation. The exclusion criteria were as follows: myopia < − 5.00 D or astigmatism > 1.50 D, any ocular disease or previous ocular surgery, other contact lens wearing history (rigid contact lens for > 4 weeks and soft contact lens for > 2 weeks), usage of drugs affecting ocular refractive status, and poor eye fixation.

All children chose Euclid (Euclid System Corporation, America) as the only type of orthokeratology contact lens and were given an explanation regarding the correct wearing method. Patients were followed-up at 1 day, 1 week, and 1 month post-orthokeratology. The collected information of both biometers from pre and 1 month post-orthokeratology were analyzed. During follow-up visit, the contact lens was in the middle location and moved along the vertical direction, with suitable fluorescent staining (dyeing) of different arc regions.

### Instruments

The new optical biometer OA-2000 (software V.1.0.R), which adopts SS-OCT to emit a laser with a wavelength of 1060 nm, exerts the B-scanning mode to measure ocular parameters [14]. Using Placido disk corneal topography, it projects nine rings to collect more than 256 points for analyzing keratometry at 2.5 mm and 3.0 mm diameter [14, 15]. During the examination, the OA-2000 uses infrared light and charge-coupled device to acquire data of AL, CT, ACD, LT, PD and CD parameters in 10 s at a time. When selecting the automatic measurement mode, it auto calibrates and automatically collects the data. Measurements were repeated if “error” or “-” signals were displayed. For each scan, 10 measurements of each parameter were acquired. Three continuous measurements were recorded.

The commonly used optical biometer IOLMaster (software V.5.4) applies PCI technology to measure ocular parameters. When six spots on the screen were all in focus, AL, K, ACD and CD parameters could be measured in turn. It sends out the infrared light at a wavelength of 780 nm to measure the AL, the distance of which (also measured by the SS-OCT biometer) is from the anterior surface of central cornea to the pigment epithelium layer of retina [16]. The signal-to-noise ratio was applied to evaluate the quality of axial length measurements. If the SNR was < 2.0, additional measurements were taken. For corneal curvature, the IOLMaster projects a slit light with 590 nm wavelength to the anterior corneal surface to form six hexagonal symmetry points and uses 1.3375 as refractive index to calculate the corneal power [17]. Five successive repeated measurements for AL, ACD were recorded, while three continuous measurements for CK and CD were noted.

### Procedures

After all children underwent a routine ophthalmological examination to meet the inclusion criteria, an experienced operator taught the subjects how to cooperate, and then took continuous measurements with the two devices in a random order. Then, the second experienced operator performed successive measurements with the new SS-OCT biometer. After the children had been wearing orthokeratology contact lenses for one month, both operators repeated the above procedures. The two instruments were calibrated before commencing the procedure. In a dimly lit room, with natural pupil condition, the subjects placed their chins on the chin rest and held their foreheads against brow band. Then, they were asked to look at the target in the front light with both eyes open. Before each measurement started, the subjects were asked to blink completely to ensure that a tear film was fully covering the cornea to provide a smooth optical surface.

Both eyes of all subjects were examined and the total time did not exceed 30 min. To avoid the effect of the correlation between eyes on results, only the right eye with qualified image according to the specification was chosen for further analysis.

Referring to previous studies and the PS program [18, 19], when two different examiners conduct three consecutive examinations respectively, a sample size of at least 24 subjects are needed to be recruited into the study to estimate the confidence interval with a power of 90%.

### Statistical analysis

The SPSS software (version 21 for Windows, IBM Corporation, USA) and MedCalc software (version 13.0, Bvba, Ostend, Belgium) were applied for statistical analysis. Kolmogorov-Smirnov test was used to assess the normality of the data distribution. A *P* value < 0.05 was considered to be statistically significant. Results were presented as mean ± standard deviation (mean ± SD).

In the present study, the term precision (repeatability and reproducibility) was used according to the definition of the International Organization for Standardization. Precision was assessed on the basis of the reproducibility limit (known also as test-retest variability) [18], the intra-subject coefficient of variation (CoV) and the intra-class correlation coefficient (ICC).

The reproducibility limit is calculated as 2.77 times the intra-subject standard deviation (S_w_) [20] and represents the 95% fluctuation range of differences between two measurements in the same individual [21]. The CoV is often expressed as a percentage and is defined as the ratio of the S_w_ to the mean value of total sample. For the above three indexes, the lower the value, the smaller is the difference and the higher is the precision [22]. The value of intra-class correlation coefficients (ICCs) is between 0 and 1.0, wherein “0” means not credible, and “1.0” means entirely credible [21]. S_w_, reproducibility limit, CoV and ICCs were calculated to assess the repeatability and reproducibility of SS-OCT.

The paired t-test and Bland-Altman plots were used to estimate the difference and agreement between two devices. For Bland-Altman plot, the x-axis stands for the mean values obtained by two instruments, while the y-axis refers to the difference between them. The 95% limit of agreement (LoA), which comes from Bland-Altman plot, represents the 95% interval of the difference between measurements from the two devices. The narrower the range of 95% LoA, the higher is the consistency [23].

## Results

Fifty-one eyes of 51 normal children (24 males and 27 females) were examined in this prospective study. The age range of subjects was 8 to 16 years (mean: 11.98 ± 2.15 years). The range of spherical equivalent was − 0.75 D to − 5.125 D (mean: − 3.37 ± 1.28 D).

### Intra-operator repeatability measurement of the new SS-OCT biometer

The new SS-OCT biometer showed remarkable intra-operator repeatability for AL, Km, CCT, ACD, LT and CD measurements on pre- and post-orthokeratology. AL showed the best intra-operator repeatability among all measured parameters. The reproducibility limit of CCT was < 12.13 μm, which meant the least repeatability as compared to other parameters. The ICC was close to 1.00 and the CoV was ≤ 1.23% for all parameters (Table 1 and Table 2).

**Table 1:** Intra-observer repeatability of ocular biological parameters measured using the new swept-source optical coherence tomography biometer in children before wearing orthokeratology lens

Parameter	Observer	Mean ± SD	S_w_	R	CoV (%)	ICC
AL (mm)	1st	25.15 ± 0.99	0.02	0.06	0.08	1.000
	2nd	25.15 ± 0.99	0.01	0.03	0.04	1.000
CCT (μm)	1st	537.59 ± 31.76	4.38	12.13	0.81	0.981
	2nd	537.46 ± 31.49	4.01	11.10	0.75	0.984
ACD (mm)	1st	3.72 ± 0.19	0.02	0.06	0.61	0.986
	2nd	3.73 ± 0.19	0.02	0.06	0.63	0.984
LT (mm)	1st	3.43 ± 0.14	0.03	0.09	0.91	0.953
	2nd	3.43 ± 0.14	0.03	0.08	0.83	0.960
Km (Φ = 2.5) (D)	1st	43.15 ± 1.45	0.10	0.28	0.23	0.995
	2nd	43.18 ± 1.44	0.08	0.22	0.18	0.997
Km (Φ = 3.0) (D)	1st	43.13 ± 1.46	0.09	0.26	0.21	0.996
	2nd	43.15 ± 1.46	0.07	0.19	0.16	0.998
CD (mm)	1st	11.96 ± 0.43	0.06	0.17	0.51	0.980
	2nd	11.96 ± 0.41	0.06	0.17	0.51	0.978

*AL*= Axial length; *CCT*= central corneal thickness; *ACD*= anterior chamber depth (corneal epithelium to lens); *LT*= lens thickness; *Km*= mean keratometry; *CD*= corneal diameter; *SD*= standard deviation; *Sw*= within-subject standard deviation; *R*= reproducibility limit (2.77 Sw); *CoV*= within-subject coefficient of variation; *ICC*= intraclass correlation coefficient

**Table 2:** Intra-observer repeatability of ocular biological parameters measured using the new swept-source optical coherence tomography biometer in children after wearing orthokeratology lens

Parameter	Observer	Mean ± SD	S_w_	R	CoV (%)	ICC
AL (mm)	1st	25.15 ± 0.99	0.01	0.03	0.04	1.000
	2nd	25.14 ± 0.99	0.01	0.04	0.06	1.000
CCT (μm)	1st	529.21 ± 31.95	4.01	11.10	0.76	0.983
	2nd	529.22 ± 32.64	4.27	11.83	0.81	0.983
ACD (mm)	1st	3.65 ± 0.22	0.02	0.06	0.64	0.983
	2nd	3.65 ± 0.23	0.05	0.13	1.23	0.962
LT (mm)	1st	3.49 ± 0.20	0.03	0.08	0.82	0.967
	2nd	3.48 ± 0.20	0.04	0.11	1.18	0.96
Km (Φ = 2.5) (D)	1st	40.81 ± 1.55	0.08	0.22	0.19	0.993
	2nd	40.80 ± 1.57	0.13	0.36	0.32	0.993
Km (Φ = 3.0) (D)	1st	41.17 ± 1.56	0.07	0.19	0.16	0.994
	2nd	41.16 ± 1.58	0.11	0.30	0.26	0.995
CD (mm)	1st	11.94 ± 0.39	0.06	0.17	0.51	0.974
	2nd	11.95 ± 0.38	0.08	0.22	0.66	0.958

*AL*= Axial length; *CCT*= central corneal thickness; *ACD*= anterior chamber depth (corneal epithelium to lens); *LT*= lens thickness; *Km*= mean keratometry; *CD*= corneal diameter; *SD*= standard deviation; *Sw*= within-subject standard deviation; *R*= reproducibility limit (2.77 Sw); *CoV*= within-subject coefficient of variation; *ICC*= intraclass correlation coefficient

### Inter-operator reproducibility measurement of the new SS-OCT biometer

The new SS-OCT biometer displayed excellent inter-operator reproducibility on pre- and post-orthokeratology. AL showed the smallest reproducibility limit with a value ≤0.02 mm and CCT presented the largest reproducibility limit with a value < 7.02 μm. For all ocular parameters, the ICC was > 0.967. Additionally, AL displayed the smallest CoV with a value ≤0.03% (Table 3).

**Table 3:** Inter-observer reproducibility of ocular biological parameters measured using the new swept-source optical coherence tomography biometer in children before and after wearing orthokeratology lens

Parameter	Status	S_w_	R	CoV (%)	ICC
AL (mm)	Before	0.01	0.02	0.03	1.000
	After	0.01	0.02	0.03	1.000
CCT (μm)	Before	2.36	6.53	0.44	0.994
	After	2.53	7.02	0.48	0.994
ACD (mm)	Before	0.02	0.06	0.54	0.988
	After	0.02	0.06	0.64	0.989
LT (mm)	Before	0.03	0.07	0.75	0.967
	After	0.02	0.07	0.71	0.985
Km (Φ = 2.5) (D)	Before	0.06	0.17	0.14	0.998
	After	0.08	0.21	0.19	0.998
Km (Φ = 3.0) (D)	Before	0.06	0.17	0.14	0.998
	After	0.06	0.18	0.15	0.998
CD (mm)	Before	0.06	0.17	0.50	0.980
	After	0.06	0.17	0.50	0.976

*AL*= Axial length; *CCT*= central corneal thickness; *ACD*= anterior chamber depth; *LT*= lens thickness; *Km*= mean keratometry; *CD*= corneal diameter; *SD*= standard deviation; *Sw*= within-subject standard deviation; *R*= reproducibility limit (2.77 Sw); *CoV*= within-subject coefficient of variation; *ICC*= intraclass correlation coefficient

### Agreement between the new SS-OCT biometer and the PCI biometer

**Fig. 1:**
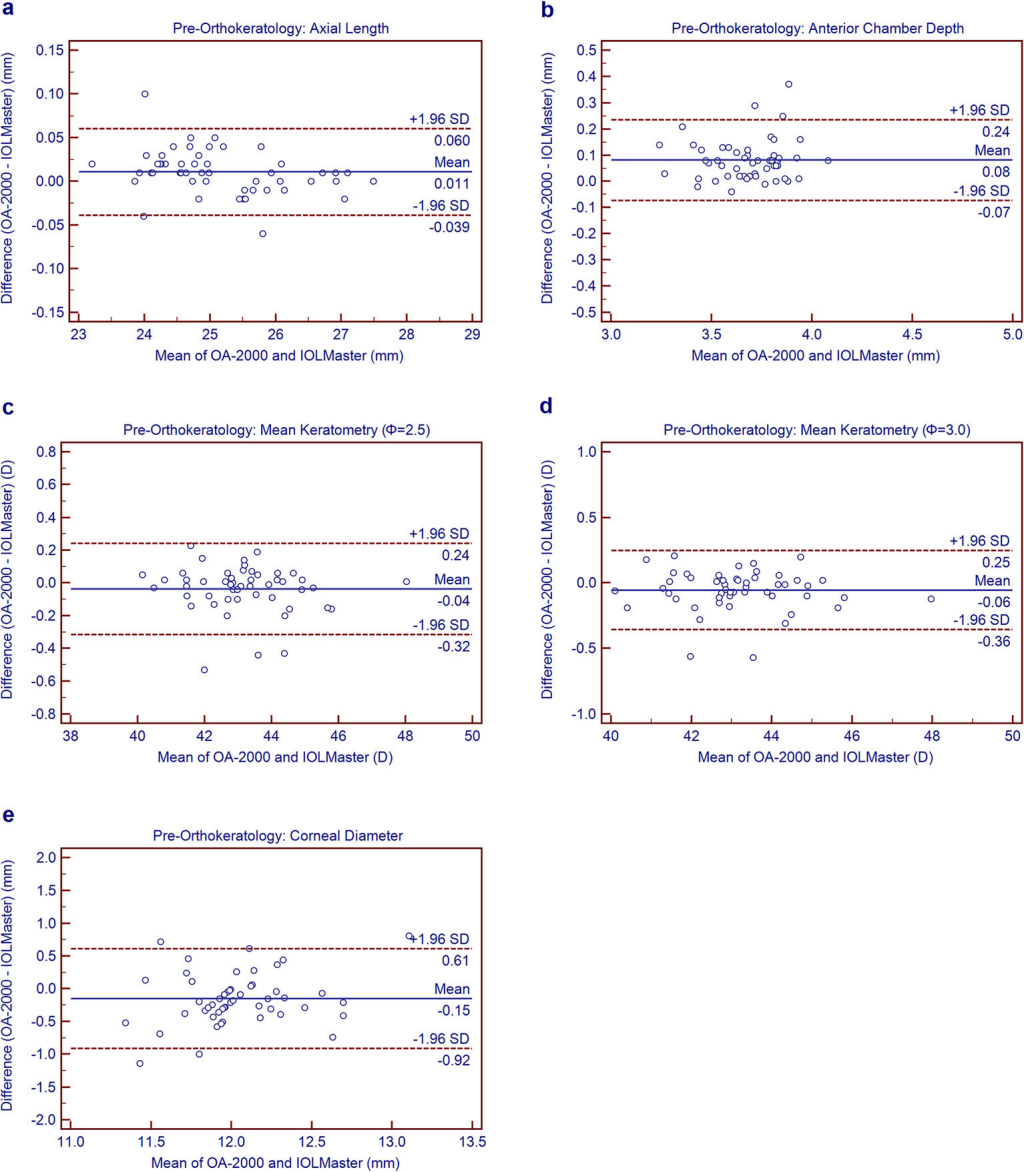
Bland-Altman plots show agreement between OA-2000 and IOLMaster for axial length (**a**), chamber depth (**b**), mean keratometry at 2.5 mm diameter (**c**), mean keratometry at 3.0 mm diameter (**d**), corneal diameter (**e**) measurement pre-orthokeratology. The solid line indicates the mean difference (bias), and the dotted lines represent the 95% limits of agreement

**Fig. 2:**
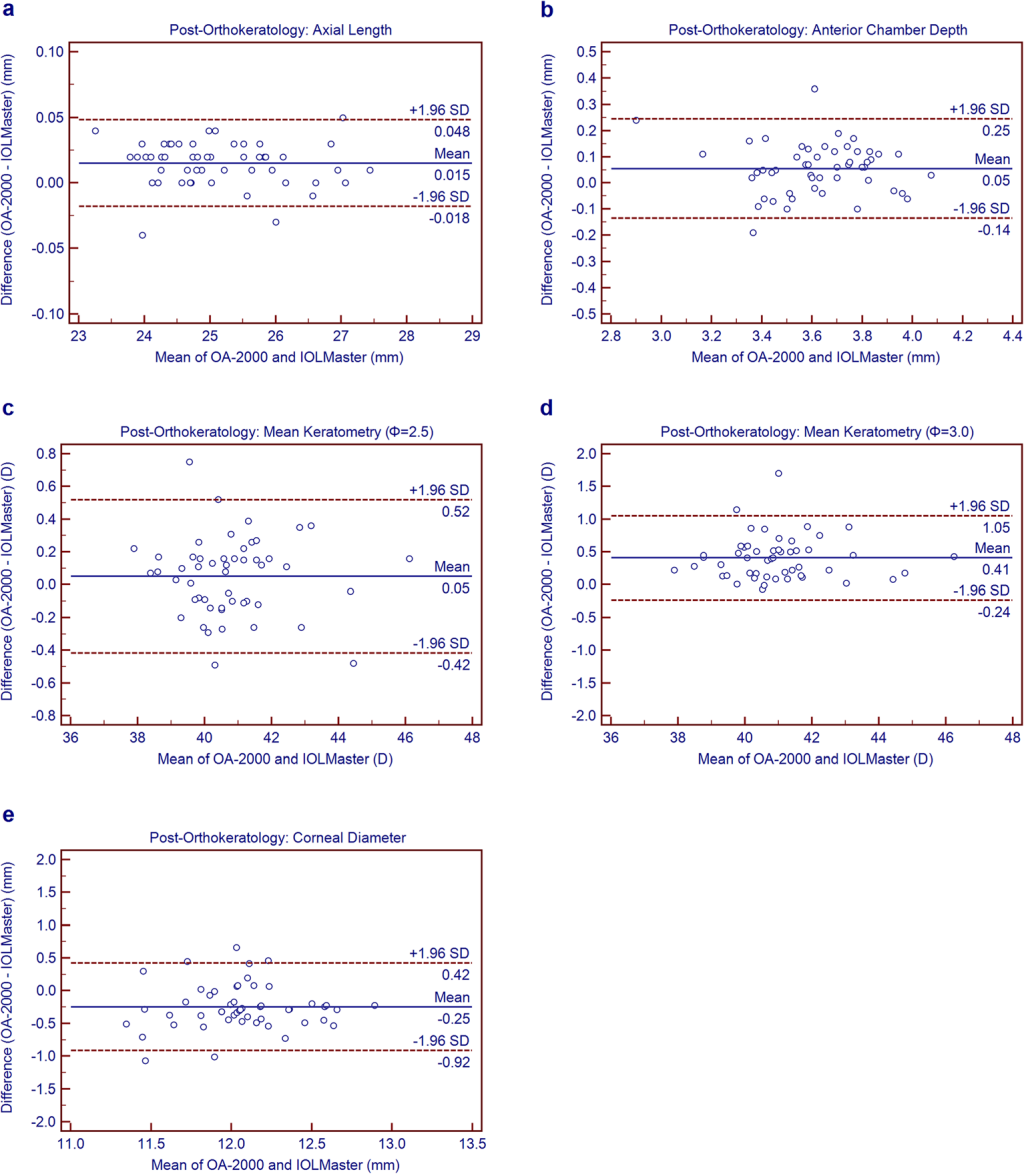
Bland-Altman plots show agreement between OA-2000 and IOLMaster for axial length (**a**), chamber depth (**b**), mean keratometry at 2.5 mm diameter (**c**), mean keratometry at 3.0 mm diameter (**d**), corneal diameter (**e**) measurement post-orthokeratology. The solid line indicates the mean difference (bias), and the dotted lines represent the 95% limits of agreement

Statistically significant differences were observed between the SS-OCT biometer and the PCI biometer for all parameters, except Km_2.5_ (Table 4). However, Figs. 1(a to e)-2(a to e) for the Bland-Altman outcomes demonstrate narrow 95% LoA for most parameters except CD, indicating high agreement between the two devices. The 95% LoA for CD were ≥ 1.34 mm, thus showing relatively poorer agreement comparing with other parameters.

**Table 4:** Comparison of ocular biological parameters measured using the new swept-source optical coherence tomography biometer and the partial coherence interferometry biometer in children before and after wearing orthokeratology lens

Device Pairings	Status	Mean ± SD	P Value	95% LoA
AL (mm)	Before	0.01 ± 0.03	0.004	−0.04 to 0.06
	After	0.01 ± 0.02	0.000	−0.02 to 0.05
ACD (mm)	Before	0.08 ± 0.08	0.000	− 0.07 to 0.24
	After	0.06 ± 0.10	0.000	−0.13 to 0.25
Km (Φ = 2.5) (D)	Before	−0.04 ± 0.14	0.070	− 0.32 to 0.24
	After	0.05 ± 0.24	0.144	−0.42 to 0.52
Km (Φ = 3.0) (D)	Before	−0.06 ± 0.15	0.013	− 0.36 to 0.25
	After	0.41 ± 0.33	0.000	−0.24 to 1.05
CD (mm)	Before	−0.15 ± 0.39	0.008	− 0.92 to 0.61
	After	−0.25 ± 0.34	0.000	− 0.92 to 0.42

*AL*= Axial length; *ACD*= anterior chamber depth; *Km*= mean keratometry; *CD* corneal diameter; *SD*= Standard deviation; *LoA*= limit of agreement

## Discussion

Inappropriate orthokeratology contact lens type or improper wearing will greatly influence the effectiveness and comfort level in children, so it is crucial to accurately examine the patient before wearing orthokeratology contact lenses. Selecting suitable orthokeratology parameters depends on the precision of measuring devices. Many studies have focused on the function of orthokeratology for myopia control [24–26], but the variation of precision measurements of the new SS-OCT optical biometers has not been reported in children wearing orthokeratology contact lenses.

As myopia is becoming increasingly common in teenagers, AL measurement plays an indispensable role in the assessment of its progression. The precision of AL measurement is currently a hot topic. The PCI biometer was reported to have high repeatability for AL measurement in children [27–30]. Kimura et al. [28] found that the repeatability for AL measurement in myopic children was ± 0.05 mm with the PCI biometer. The PCI biometer provided precise measurements of AL parameters in normal children in a study conducted by Quinn et al. [27], Hussin et al. [31], and Carkeet et al. [32] who also demonstrated that the PCI biometer showed narrow 95% LoA for repeatability of AL measurement in children. Chan et al. [33] indicated that the PCI biometer showed high repeatability of AL measurement in children undergoing orthokeratology, with narrow between-measurement 95% LoA of − 0.04 to 0.05 mm.

Powerful tissue penetration, high scanning speed and short inspection time are the unique advantages of SS-OCT biometer for AL examination in children with poor cooperation. Using three SS-OCT biometers to measure AL for 119 cataract patients, Huang et al. [34] found excellent repeatability for these instruments with low reproducibility limit (≤ 0.06 mm) and CoV (≤ 0.10%) values. In this study, before and after orthokeratology, AL measurement showed the smallest reproducibility limit with a value ≤0.02 mm, i.e. the SS-OCT biometer displayed the highest repeatability and reproducibility for AL measurements. Huang et al. [10] found that the SS-OCT showed excellent precision with S_w_ ≤ 0.03 mm in measuring the AL of adults. Wang et al. [29] demonstrated that the SS-OCT biometer displayed higher ICC and smaller CoV for AL measurement in cataract patients.

The IOLMaster 700 is another SS-OCT optical biometer, which has exhibited outstanding repeatability and reproducibility for AL measurements in different groups of people. Utilizing the IOLMaster 700 in cataract patients, Srivannaboon et al. [35] found that the ICC of AL measurement was up to 1.0, which suggested excellent precision. Similar outcomes were also reported by Manael et al. [36] and Teresa et al. [37] in normal eyes with clear lens.

Before and after orthokeratology, the absolute values for AL between the SS-OCT biometer and PCI biometer were < 0.10 mm (Figs. 1a and 2a), indicating high agreement between the two instruments. Huang et al. [10] and Gao et al. [38] also indicated high agreement between the SS-OCT biometer and IOLMaster or Lenstar in measuring AL in healthy eyes. In contrast, Lenhart et al. [39] showed that the 95% confidence interval between IOLMaster and immersion ultrasound for AL was wide with a value of 0.30 mm in children, which showed a poorer agreement than the results of the current study. Comparing three instruments for measuring AL in cataract patients, Goebels et al. [7] and McAlinden et al. [11] reported that the SS-OCT biometer provided more accurate results, and the difference between the three instruments was narrow.

When Huang et al. [10] used two devices to measure anterior ocular segment parameters, the SS-OCT biometer showed high precision in keratometry with a reproducibility limit < 0.19 D. Goebels et al. [7] also obtained good results with the same SS-OCT biometer. In this study, before and after orthokeratology, the reproducibility limit for Km_2.5_ and Km_3.0_ were < 0.35 D and the CoV did not exceed 0.31%, demonstrating high repeatability and reproducibility. Similar to the new SS-OCT biometer, the Cassia SS-1000 (Tomey, Nagoya, Japan) uses the SS-OCT technology to gain three-dimensional anterior ocular segment images and was also used by Lee et al. [40] for keratometry measurement in post-refractive surgery eyes, which showed that the ICC were > 0.927, suggesting excellent repeatability.

Before orthokeratology, the mean value of Km_2.5_ was 43.15 ± 1.45 D, which was slightly higher than Km_3.0_ (43.13 ± 1.46 D). Similar results were obtained by Huang et al. [10, 29] when using the SS-OCT biometer in healthy adults and cataract patients. In contrast, after orthokeratology, the mean value of Km_2.5_ was 40.81 ± 1.55 D, which was smaller than Km_3.0_ in another study. This difference was related to the mechanism of orthokeratology [41]. While wearing orthokeratology contact lenses, the compression played a major role in the central cornea, and the closer the lens is to the central area, the more obvious is the compression effect. Hence, the center of the cornea became flatter and the periphery steeper.

Judging agreement between the two devices using the Bland-Altman plots (Figs. 1c-d and 2c-d), the 95% LoA of Km_2.5_ after orthokeratology were 0.68 times larger than before orthokeratology. However, the 95% LoA of Km_3.0_ increased up to 1.29 D, which was 1.11 times larger than before, i.e. after orthokeratology, the Km_3.0_ values showed poorer agreement between the two instruments than Km_2.5_. The corneal curvature with a diameter of 2.5 mm can more accurately reflect the change of corneal curvature after wearing orthokeratology. Hence, we recommend relying on Km_2.5_ to accurately monitor the changes of keratometry during the follow-up of orthokeratology so as to better guide the adjustment of orthokeratology size and improve the visual acuity of children in the daytime.

Using the SS-OCT optical biometer to collect CCT, ACD and LT parameters in healthy adults, Grulkowski et al. [42] indicated that their ICC were > 0.99. In the present study, before and after orthokeratology, the CCT, ACD and LT measurements by the new SS-OCT biometer showed excellent repeatability and reproducibility with low CoV and high ICC values. Sahin et al. [43] found that the ICC of ACD measurement in children by Lenstar was > 0.991, which was similar to our findings.

Carkeet et al. [32] compared IOLMaster with A-scan for measuring ACD in children, and the fluctuation ranged from − 0.28 mm to 0.46 mm. In our Bland-Altman plots (Figs. 1b and 2b), the agreement between the two devices for ACD measurement was high with smaller values (≤ 0.38 mm), which was narrower than the above report.

Huang et al. [10] found that the OA-2000 biometer showed excellent precision with low reproducibility limit (≤ 0.14 mm) for CD measurement. In the present study, the CD measurement displayed a high ICC value, which also suggested good precision measurement in children. However, the maximum of the absolute values of 95% LoA for CD measurement was 1.53 mm, revealing poor agreement between the SS-OCT and PCI optical biometers (Figs. 1e and 2e). Similar conclusions were reported by others. Kongsap et al. [44] found a weak correlation between OA-2000 and IOLMaster 500 for CD measurement in cataract patients. In a meta-analysis, Huang et al. [45] found a statistically significant difference in CD parameter between the IOLMaster and Lenstar. Reviewing the agreement between eight different devices for CD measurement, Alberto et al. [46] concluded that the fluctuations between them were too wide for interchangeable use in clinical practice.

The main limitation of this study was the lack of data on longer duration of orthokeratology contact lens use. Also, the precision measurements are needed for aphakic eyes, vitreous hemorrhage, keratoconus and post-refractive surgery patients.

## Conclusions

In summary, after orthokeratology, the SS-OCT biometer showed high repeatability and reproducibility for all measurements, which were similar to before orthokeratology. It is suggested that orthokeratology does not affect the repeatability and reproducibility of measurements by the SS-OCT optical biometer. This instrument can be used as a better tool for children’s ocular examination in the clinic. Except for the CD measurement, the SS-OCT and the PCI optical biometers showed high agreement. Wearing orthokeratology contact lenses influenced the agreement between SS-OCT and PCI biometers for Km_3.0_ measurements, but had no effect for AL, Km_2.5_, ACD and CD measurements.

## Data Availability

All data generated or analyzed during this study are included in this published article.

## Abbreviations

ACD: Anterior chamber depth
AL: Axial length
CCT: Central corneal thickness
CD: Corneal diameter
CoV: Coefficient of variation
CT: Corneal thickness
ICC: Intra-class correlation coefficient
K: Keratometry
Km2.5: Mean keratometry at 2.5 mm diameter
Km3.0: Mean keratometry at 3.0 mm diameter
LoA: Limit of agreement
LT: Lens thickness
Mean ± SD: Mean ± standard deviation
PCI: Partial coherence interferometry
PD: Pupil diameter
SS-OCT: Swept-source optical coherence tomography
S_w_: Intra-subject standard deviation

## References

[1] Kang P, Swarbrick H (2016). New perspective on myopia control with orthokeratology. Optom Vis Sci.

[2] Swarbrick HA (2006). Orthokeratology review and update. Clin Exp Optom.

[3] Santodomingo-Rubido J, Villa-Collar C, Gilmartin B, Gutiérrez-Ortega R. Myopia control with orthokeratology contact lenses in Spain: refractive and biometric changes. Invest Ophthalmol Vis Sci. 2012;53(8):5060–5. 10.1167/iovs.11-8005 22729437

[4] Kakita T, Hiraoka T, Oshika T (2011). Influence of overnight orthokeratology on axial elongation in childhood myopia. Invest Ophthalmol Vis Sci.

[5] Na KS, Yoo YS, Hwang HS, Mok JW, Kim HS, Joo CK (2016). The influence of overnight orthokeratology on ocular surface and meibomian glands in children and adolescents. Eye Contact Lens.

[6] Cheung SW, Cho P, Chui WS, Woo GC (2007). Refractive error and visual acuity changes in orthokeratology patients. Optom Vis Sci.

[7] Goebels S, Pattmöller M, Eppig T, Cayless A, Seitz B, Langenbucher A. Comparison of 3 biometry devices in cataract patients. J Cataract Refract Surg. 2015;41(11):2387–93. 10.1016/j.jcrs.2015.05.028 26703487

[8] Shammas HJ, Chan S (2010). Precision of biometry, keratometry, and refractive measurements with a partial coherence interferometry-keratometry device. J Cataract Refract Surg.

[9] Kunavisarut P, Poopattanakul P, Intarated C, Pathanapitoon K (2012). Accuracy and reliability of IOL master and A-scan immersion biometry in silicone oil-filled eyes. Eye (Lond).

[10] Huang J, Savini G, Hoffer KJ, Chen H, Lu W, Hu Q (2017). Repeatability and interobserver reproducibility of a new optical biometer based on swept-source optical coherence tomography and comparison with IOLMaster. Br J Ophthalmol.

[11] McAlinden C, Wang Q, Gao R, Zhao W, Yu A, Li Y (2017). Axial length measurement failure rates with biometers using swept-source optical coherence tomography compared to partial-coherence interferometry and optical low-coherence interferometry. Am J Ophthalmol.

[12] Koepsell D, Brinkman WP, Pont S (2015). Human participants in engineering research: notes from a fledgling ethics committee. Sci Eng Ethics.

[13] Read SA, Collins MJ (2009). Diurnal variation of corneal shape and thickness. Optom Vis Sci.

[14] Holzer MP, Mamusa M, Auffarth GU (2009). Accuracy of a new partial coherence interferometry analyser for biometric measurements. Br J Ophthalmol.

[15] Chen YA, Hirnschall N, Findl O (2011). Evaluation of 2 new optical biometry devices and comparison with the current gold standard biometer. J Cataract Refract Surg.

[16] Santodomingo-Rubido J, Mallen EA, Gilmartin B, Wolffsohn JS (2002). A new non-contact optical device for ocular biometry. Br J Ophthalmol.

[17] Lopez de la Fuente C, Sanchez-Cano A, Segura F, Pinilla I (2014). Comparison of anterior segment measurements obtained by three different devices in healthy eyes. Biomed Res Int.

[18] McAlinden C, Khadka J, Pesudovs K (2015). Precision (repeatability and reproducibility) studies and sample-size calculation. J Cataract Refract Surg.

[19] Huang J, Savini G, Wu F, Yu X, Yang J, Yu A (2015). Repeatability and reproducibility of ocular biometry using a new noncontact optical low-coherence interferometer. J Cataract Refract Surg.

[20] Bland JM, Altman DG (1996). Measurement error. BMJ..

[21] Savini G, Barboni P, Carbonelli M, Hoffer KJ (2011). Repeatability of automatic measurements by a new Scheimpflug camera combined with Placido topography. J Cataract Refract Surg.

[22] Aramberri J, Araiz L, Garcia A, Illarramendi I, Olmos J, Oyanarte I (2012). Dual versus single Scheimpflug camera for anterior segment analysis: precision and agreement. J Cataract Refract Surg.

[23] McAlinden C, Khadka J, Pesudovs K (2011). Statistical methods for conducting agreement (comparison of clinical tests) and precision (repeatability or reproducibility) studies in optometry and ophthalmology. Ophthalmic Physiol Opt.

[24] Santodomingo-Rubido J, Villa-Collar C, Gilmartin B, Gutiérrez-Ortega R, Sugimoto K (2017). Long-term efficacy of orthokeratology contact lens wear in controlling the progression of childhood myopia. Curr Eye Res.

[25] Chen C, Cheung SW, Cho P (2013). Myopia control using toric orthokeratology (TO-SEE study). Invest Ophthalmol Vis Sci.

[26] Hiraoka T, Kakita T, Okamoto F, Oshika T (2015). Influence of ocular wavefront aberrations on axial length elongation in myopic children treated with overnight orthokeratology. Ophthalmol..

[27] Quinn GE, Francis EL, Nipper KS, Flitcroft DI, Ying GS, Rees RC (2003). Highly precise eye length measurements in children aged 3 through 12 years. Arch Ophthalmol.

[28] Kimura S, Hasebe S, Miyata M, Hamasaki I, Ohtsuki H (2007). Axial length measurement using partial coherence interferometry in myopic children: repeatability of the measurement and comparison with refractive components. Jpn J Ophthalmol.

[29] Wang W, Miao Y, Savini G, McAlinden C, Chen H, Hu Q (2017). Precision of a new ocular biometer in eyes with cataract using swept source optical coherence tomography combined with Placido-disk corneal topography. Sci Rep.

[30] Yağcı R, Güler E, Kulak AE, Erdoğan BD, Balcı M, Hepşen İF (2015). Repeatability and reproducibility of a new optical biometer in normal and keratoconic eyes. J Cataract Refract Surg.

[31] Hussin HM, Spry PG, Majid MA, Gouws P (2006). Reliability and validity of the partial coherence interferometry for measurement of ocular axial length in children. Eye (Lond)..

[32] Carkeet A, Saw SM, Gazzard G, Tang W, Tan DT (2004). Repeatability of IOLMaster biometry in children. Optom Vis Sci.

[33] Chan B, Cho P, Cheung SW (2006). Repeatability and agreement of two A-scan ultrasonic biometers and IOLMaster in non-orthokeratology subjects and post-orthokeratology children. Clin Exp Optom..

[34] Huang J, Chen H, Li Y, Chen Z, Gao R, Yu J (2019). Comprehensive comparison of axial length measurement with three swept-source OCT-based biometers and partial coherence interferometry. J Refract Surg.

[35] Srivannaboon S, Chirapapaisan C, Chonpimai P, Loket S (2015). Clinical comparison of a new swept-source optical coherence tomography-based optical biometer and a time-domain optical coherence tomography-based optical biometer. J Cataract Refract Surg.

[36] Garza-Leon M, Fuentes-de la Fuente HA, Garcia-Treviño AV. Repeatability of ocular biometry with IOLMaster 700 in subjects with clear lens. Int Ophthalmol. 2017;37(5):1133–8. 10.1007/s10792-016-0380-7 27770390

[37] Ferrer-Blasco T, Dominguez-Vicent A, Esteve-Taboada JJ, Aloy MA, Adsuara JE, Montés-Micó R (2017). Evaluation of the repeatability of a swept-source ocular biometer for measuring ocular biometric parameters. Graefes Arch Clin Exp Ophthalmol.

[38] Gao R, Chen H, Savini G, Miao Y, Wang X, Yang J (2017). Comparison of ocular biometric measurements between a new swept-source optical coherence tomography and a common optical low coherence reflectometry. Sci Rep.

[39] Lenhart PD, Hutchinson AK, Lynn MJ, Lambert SR (2010). Partial coherence interferometry versus immersion ultrasonography for axial length measurement in children. J Cataract Refract Surg.

[40] Lee YW, Choi CY, Yoon GY (2015). Comparison of dual rotating Scheimpflug-Placido, swept-source optical coherence tomography, and Placido-scanning-slit systems. J Cataract Refract Surg.

[41] Maseedupally V, Gifford P, Lum E, Swarbrick H (2013). Central and paracentral corneal curvature changes during orthokeratology. Optom Vis Sci.

[42] Grulkowski I, Liu JJ, Zhang JY, Potsaid B, Jayaraman V, Cable AE (2013). Reproducibility of a long-range swept-source optical coherence tomography ocular biometry system and comparison with clinical biometers. Ophthalmol..

[43] Sahin A, Gursoy H, Basmak H, Yildirim N, Usalp Z, Çolak E (2011). Reproducibility of ocular biometry with a new noncontact optical low-coherence reflectometer in children. Eur J Ophthalmol.

[44] Kongsap P (2016). Comparison of a new optical biometer and a standard biometer in cataract patients. Eye Vis (Lond).

[45] Huang J, McAlinden C, Huang Y, Wen D, Savini G, Tu R (2017). Meta-analysis of optical low-coherence reflectometry versus partial coherence interferometry biometry. Sci Rep.

[46] Dominguez-Vicent A, Perez-Vives C, Ferrer-Blasco T (2016). Device interchangeability on anterior chamber depth and white-to-white measurements: a thorough literature review. Int J Ophthalmol.

